# Associations Between Rate of Force Development Metrics and Throwing Velocity in Elite Team Handball Players: a Short Research Report

**DOI:** 10.2478/v10078-011-0059-0

**Published:** 2011-10-04

**Authors:** Mário C. Marques, Francisco J. Saavedra, Catarina Abrantes, Felipe J. Aidar

**Affiliations:** 1 Department of Sport Sciences, University of Beira Interior (UBI), Covilhã, Portugal; 2 Research Centre for Sport Sciences, Health and Human Development (CIDESD), Vila Real, Portugal; 3 Department of Sport Sciences, University of Trás-os-Montes and Alto Douro (UTAD), Vila Real, Portugal

**Keywords:** rate of force development, velocity, throwing, elite, team handball

## Abstract

Performance assessment has become an invaluable component of monitoring participant’s development in distinct sports, yet limited and contradictory data are available in trained subjects. The purpose of this study was to examine the relationship between ball throwing velocity during a 3-step running throw in elite team handball players and selected measures of rate of force development like force, power, velocity, and bar displacement during a concentric only bench press exercise in elite male handball players. Fitteen elite senior male team handball players volunteered to participate. Each volunteer had power and bar velocity measured during a concentric only bench press test with 25, 35, and 45 kg as well as having one-repetition maximum strength determined. Ball throwing velocity was evaluated with a standard 3-step running throw using a radar gun. The results of this study indicated significant associations between ball velocity and time at maximum rate of force development (0, 66; p<0.05) and rate of force development at peak force (0,56; p<0.05) only with 25kg load. The current research indicated that ball velocity was only median associated with maximum rate of force development with light loads. A training regimen designed to improve ball-throwing velocity in elite male team handball players should emphasize bench press movement using light loads.

## Introduction

Team Handball (TH) consists of intense, intermittent activities such as running, sprinting, jumping as well as regular throwing, hitting, blocking, and pushing between players. In addition to technical and tactical skills, it has been argued that one of the key skills necessary for success in team handball is throwing performance (Gorostiaga et al., 2006; Marques and González-Badillo, 2006). The technique of motion and the fitness level can be improved by the training process, others variables like strength and power can determine throwing ball velocity (TBv). Although muscular strength and power out put have been reported to be associated with throwing velocity, limited data have been published with elite male handball players.

To our best knowledge, few studies have examined the relationship between ball throwing velocity in elite team handball players with distinct strength metrics during muscle contractions of the upper-extremity in concentric only bench press exercise (Gorostiaga et al., 2006; Marques et al., 2007). Other investigations have used isokinetic and isometric tests as indices of strength, but single joint actions not specific assessment strategies. In other words, using a strength test with constant speed (i.e., isokinetic) or a test where muscle action is not accompanied by motion (i.e., isometric) may be less suitable for athletics than a test that allows for variable speeds throughout the range of motion (i.e., isotonic). The bench press exercise was chosen because it seems most specific to overhand throwing technique. Thus, using a multi-joint exercise such as the bench press test should be advantageous when exploring for relationships with a dynamic movement such as throwing.

None of the previous studies examined throwing velocity with rate of force development metrics such us force, power, velocity, and bar displacement during a concentric only bench press exercise in elite male handball players. Therefore, the aim of this study was to determine the relationships between throwing ball velocity in elite team handball players and selected measures of rate of force development like force, power, velocity, and bar displacement during a concentric only bench press exercise in elite male handball players. Examination of these relationships could be of great importance for the optimal development of resistance training programs to improve handball-throwing performance in professional handball athletes.

## Material and Methods

A group of 15 senior elite male team handball players volunteered to participate in the study (average age: 23, range 19–28), which included five Portuguese international players. Participants were trained by the same coach and for the same club team for the 2 years prior to testing. The team has been rated as one of the best Portuguese elite team handball squads. Before commencing the study, players had a physical examination by the team physician, and each was cleared of any medical disorders that might limit full participation in the investigation. Subjects were required to sign an informed consent form prior to the study that had been approved by the Institutional Review Committee Board of the local Committee for Medical Research Ethics and current Portuguese law and regulations, and was carried out according to the Helsinki Declaration.

The concentric only bench press exercise was used to simultaneously assess dynamic strength, power, and bar velocity and was performed after the evaluation of throwing velocity. Ball throwing velocity was evaluated on an indoor team handball court by an over arm throw using a 3-step running throw, which is commonly performed during team handball. After a 10 minute standardized warm up the subjects were instructed to throw a standard handball (mass 0,48 kg; circumference 58 cm) for maximal velocity at a standard goal, using their preferred throwing hand and own throwing technique. Players were allowed only a 3-step preparatory run, and were required to release the ball behind the 9 meter line. Each subject executed five throws with two minutes rest between each trial. An average of the four throws with the greatest velocity were used for analysis. The coaches supervised the entire throwing test to ensure that the subjects were using an overarm throwing technique regularly used in handball. The ball throwing velocity (TBv) was determined using a Doppler radar gun (Sports Radar 3300, Sports Electronics Inc.), with ± 0.1 km/h accuracy within a field of 10 degrees from the gun. The Doppler radar gun was located behind a wooden target (Ø: 60 centimetres) that had a hole in the middle in order to permit optical contact with the ball and the player. Intraclass correlation coefficient (ICC) for TBv was ICC = 0.95 (95% confidence interval: 0.91–0.96) and a coefficient of variation (CV) of 3.5%.

Dynamic strength was assessed with a 1-repetition concentric only maximal bench press action (1RM) using a free-weight barbell machine. To begin the test and with the help of two coaches, the bar was positioned on the athlete’s chest, and was required to remain there for about one second prior to initiating movement in an effort to minimize any countermovement effect on any of the performance indices. Next, each athlete was instructed to perform a concentric only action from this starting position, as quickly as possible, until full extension of the elbows occurred. A trial was discounted if there appeared to be an initial countermovement of the bar, if the lower back and/or buttocks were elevated off the bench, or if an athlete failed to achieve full elbow extension. The 1RM showed an ICC of 0.91 range (95% interval: 0.72–0.98) and a CV of 6.7%. All participants used an initial weight of 25 kg, which was subsequently increased by increments of 10 or 5 kg for each trial until an individual could not execute a successful lift. Subjects performed a single repetition at each absolute load, with at least 3 minutes of rest between all trials to reduce the likelihood of fatigue. The last bearable load was determined as the 1RM. Bar displacement, average velocity (m•s^−1^), and average power (W) were recorded by attaching a rotary encoder to the end of the bar. The rotary encoder recorded the position and direction of the bar to within an accuracy of 0.0002 m. Customized software (JLML I+D, Madrid, Spain) was used to calculate average power for each repetition of the bench press performed throughout the whole range of motion as a most representative mechanical parameter associated with a contraction cycle of arm extensor muscles participating in the bench press (i.e., elbow and shoulder joints) performance. The reproducibility of the measurements has been reported elsewher (Marques et al., 2007). For testing, absolute (i.e., 25, 35, and 45 kg) rather than relative 1RM loads were used, as reported previously. Only the first three trials were taken for analysis because power declined significantly (p= 0.032) after the third trial (45 kg).

### Statistical Analyses

Standard statistical methods were used for the calculation of means and standard deviations. The Pearson product moment correlation coefficient was used to examine the association between strength, power, and velocity from the concentric only bench press exercise at each absolute load with ball throwing velocity. Statistical significance was accepted at p ≤0.05 for all analysis.

## Results

In brief, although previous studies indicated that TBv was significant related with distinct kinetics parameters during the concentric bench press, the present research indicated that TBv was only fairly associated with time at maximum rate of force development (r = 0.66; p<0.05) and rate of force development at peak force (r = 0.56; p<0.05) with light loads (25 kg).

## Discussion

The goal of this study was to determine the relationships between throwing ball velocity in elite team handball players and selected measures of rate of force development like force, power, velocity, and bar displacement during a concentric only bench press exercise in elite male handball players. To our best knowledge, this is the first study attempting to examine this issue with so much extent metrics measured with a linear transducer that can better explain throwing performance in a group of trained athlete’s population as the one presented here. The major findings of this study were the non significant correlations between throwing velocity and maximum rate of rate of force. Yet, the current experiment could observe significant relationships between time and force at rate of force development but only with light external loads.

The rate of force development has been one of the most important variables to explain performance in activities where great acceleration is required (Marques et al., 2007). This can be related to the fact that the greater the RFD, the higher will be the power and the force generated against the same load (González-Badillo and Marques, 2010). In most sports activities, the RFD is strongly related to performance abilities such as sprinting, jumping, and throwing (Kawamori et al., 2006), in which force production time is very small (between the 100 and the 300 ms) (Murphy and Wilson, 1996). For example, previous reports examining the relationship between the rate of force development and jump performance have provided equivocal findings, with some studies reporting a relationship (Matavuj et al., 2001), and others failing to observe a positive association (Young and Bilby, 1993), corroborating our results.

Nevertheless, more noticeable was the significant predictive value of the percentage of peak force (r= 0.613) at RFD_max._. However, no prior study has included this parameter for any kind of analysis. Here, the TBv is higher when the RFD_max_ is produced sooner and the peak force produces superior values (i.e. the peak force attained at RFD_max_ tends to be smaller). Therefore, this result seems to indicate that if the percentage of the maximum peak of force applied at the moment of attaining the RFD_max_ is reduced, the height of the jump tends to be greater.

Neither the velocity nor displacement at RFD_max_ during the concentric bench press showed significant a correlation with the throwing performance, except for the velocity attained at RFD_max_ using a 45 kg external load. This lack of significant association is probably explained “statistically”, since both velocity and displacement attained at the RFD_max_ are very small and very similar in all subjects. This lack of changeability reduces the possibility of a high correlation between them. What differentiate the throwing velocity are the force produced and the time taken in reaching it, not the displacement or the velocity by which the RFD_máx_ is reached (Marques, 2007). To our knowledge, no prior study has analysed these two variables as possible predictors of vertical jump height in a sample of resistance-trained participants. Yet, time taken to reach the RFD_max_ showed a significant correlation (r=−0.57) with throwing performance, but also during 45 kg external load assessment. This finding reinforces the importance of the rate of force development in those sports in which is necessary to reach high accelerations over very short times, especially considering that, in improving performance (i.e. throwing velocity ), less time is available to apply force.

It is difficult to compare the results of these studies because they differ markedly in a number of factors, including the method of measurement. Several studies used isometric techniques whereas others employed isoinertial methods (Abernethy et al., 1995). A problem with the use of isometric tests is that they only represent the strength at the specific angle measured. Furthermore, in power sports domain not many of the movements are isometric during the throwing action, and therefore it is not natural to test isometric strength in relationship with a high-velocity movement such as throwing (Murphy and Wilson, 1996). In addition, although electromyography data were not reported in the current investigation, the past literature indicates that different motor unit activation patterns exist between dynamic and isometric muscle actions; this could explain, in part, the poor correlations found in the previous investigations between the dynamic and isometric RFD_max_ (Marques et al., 2007).

In brief, as predictors, it is important that time, force at the rate of force development and the percentage of peak force produced at the maximum rate of force during the concentric phase be maintained with high values of correlation to throwing velocity. This research has been concerned to measure the different variables with instrumental rigor and a high degree of reliability. Moreover, this study was conduced in a population of trained sportsmen, thus permitting greater confidence in the results.

## Figures and Tables

**Table 1:** Linear relationships between throwing velocity and distinct measures of bench press strength on linear transducer

	r			p value		
Paramters	25 kg	35 kg	45 kg	25 kg	35 kg	45 kg
	
RFD_max._ (N · s^−1^)	Ns					
Time at RFD_max_ (ms)	r= 0.571^*^			p= 0.042		
Force at RFD_max_ (N)	Ns					
Power at RFD_máx._ (W)	Ns					
Velocity at RFD_max_ (m.s^−1^)	Ns		r= 0.56^*^			p= 0.043
Displacement at RFD_max_ (m)	Ns					
% of PF at RFD_max_ (N)	r= 0.613^*^			p= 0.026		
RFD at PF (N·s^−1^)	Ns					

Significance:

* p<0.05; Ns: non-significant;

RFD_Max_: maximum rate of force development; PF: peak force
